# Notes and News

**Published:** 1896-08-08

**Authors:** 


### Notes and News.

(Collected and Communicated.)

The question of a site for the new infirmary at Newcastle is again being discussed, but the difficulty appears to be as far from solution as ever.

The German Government were near passing a law which prohibited the plea of incurable insanity as a reason for matrimonial separation. At the last reading of the Bill a very narrow majority enabled the plea to hold good.

The Jenner Centenary has been celebrated in Japan. In Sweden the occasion has also been celebrated. In Sweden vaccination was early made obligatory, and now it is proposed to require re-vaccination also in that country.

The late Miss Elizabeth Mills, of Stanton, has bequeathed £500 to the Gloucester Infirmary, £500 to the Cheltenham Hospital, and the same sum each to the Dispensary, Tewkesbury, the Children's Hospital, Gloucester, the Winchcomb Rural Hospital, Dr. Barnardo's Home, and £200 to the Rural Hospital, Tewkesbury.

A good deal of discussion has been taking place at the Edinburgh Royal Infirmary relative to the proposed expenditure of £600 for a bathing department containing Turkish, douche, vapour, and sulphur baths for the use of patients. The scheme was adopted after some opposition on the ground of unnecessary expense.

The celebrated Barium Springs at Llangammarch Wells, Central Wales, are attracting visitors in large numbers just now. The properties of these waters have received especial attention lately owing to the prominence given to the Schott treatment of heart disease. This treatment is carried out under medical supervision at Llangammarch Wells. The guests at the Lake Hotel include Sir Spencer Wells and his daughter, Dr. and Mrs. Davis, Dr. Thorne of Leamington, Dr. W. Black Jones, Miss Barclay, and Miss Broughton.

An anonymous donor has sent a contribution of £500 towards the Quarier Homes for Consumption in Scotland.

The Tottenham Hospital has received the generous gift of £1,000 from the widow of Mr. Samuel Morley, who in his lifetime took a great interest in the institution.

A bazaar held at Camden in aid of the Carrington Convalescent Home, situate some two miles out of that town and some forty-two from Sydney, realised a sum of £130 net. This will form a nucleus for assisting the committee to carry out an improved water supply.

The Local Government Board has sanctioned the purchase of the Highgate Hill Smallpox Hospital by the Islington Guardians, to be converted into an infirmary. The Metropolitan Asylums Board, which owns the property, is to receive £53,000 for it, which sum will be provided by the London County Council at £2 17s. 6d. per cent.

The International Homœopathic Congress, which meets once in every five years, held its meetings during the present week in the Queen's Hall. The president's reception took place on Monday evening. The proceedings were varied by vocal and instrumental music, a demonstration of the Röntgen photography, and an exhibition of the nursing models from the London Homœopathic Hospital. Papers on medical and surgical subjects were read and discussed.

Fourteen old sailors have been admitted this year into the Home of the Royal Alfred Aged Merchant Seamen's Institution, and 25 others have been granted pensions of £12 a year. The present number of inmates of the Belvedere Home is 100, and the out-pensioners number 236. The benefits of the society are eagerly sought for, and the names of 260 aged seamen are in the books awaiting assistance. The society has had to borrow to defray the expenses of the past year, and consequently the benefits they can bestow have to be very limited.

We learn that the new children's branch of the Metropolitan Convalescent Institution at Broadstairs will be opened for the reception of patients on Friday, the 24th ult. This branch of the institution, which was situated for some years at Kingston Hill, was removed to temporary premises at Herne Bay in 1892, in order that the patients might have the benefit of sea air. The home at Broadstairs, which stands in five acres of ground, quite close to the sea-shore, has accommodation for 100 children, and contains special wards for surgical cases. It has been erected and furnished at a total cost of about £15,000, towards which only a little over £10,000 has been raised, and an earnest appeal is made to the benevolent for donations towards the amount still required. Children will be admitted upon the recommendation of subscribers, for a period of three weeks, entirely free of charge, the only expense being their railway fare to and from the institution. Subscriptions and donations will be gratefully acknowledged by the treasurers, Sir John Tilley, K.C.B., and Mr. Richard B. Wade; or by the secretary, Mr. Alexander Hayes, 32, Sackville Street, W.

